# Editorial: Molecular Mechanisms of Autophagy in Cancer

**DOI:** 10.3389/fcell.2022.918511

**Published:** 2022-06-17

**Authors:** Magali Humbert, Silvia Vega-Rubin-de-Celis, Guillermo Velasco, Yongjie Wei

**Affiliations:** ^1^ HemostOD SA, Lausanne, Switzerland, University of Lausanne, Lausanne, Switzerland; ^2^ Institut for Cell Biology (Cancer Research), Essen University Hospital, Essen, Germany; ^3^ Complutense University Madrid, Madrid, Spain; ^4^ Instituto de Investigación Sanitaria San Carlos (IdISSC), Madrid, Spain; ^5^ Cancer Hospital and Research Institute, Guangzhou Medical University, Guangzhou, China

**Keywords:** autophagy, cancer, therapy, CMA, biomarkers

**FIGURE 1:**
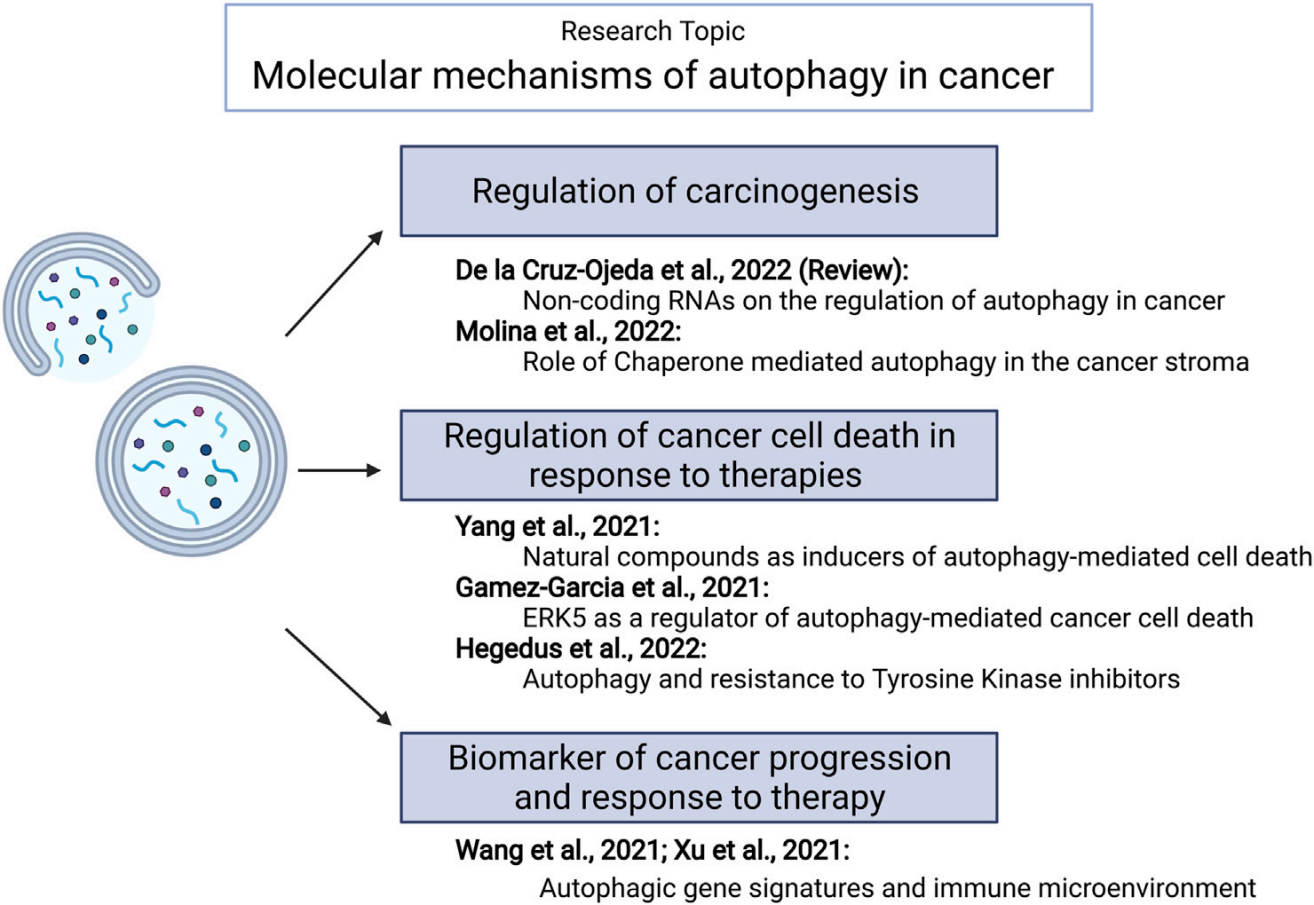
Summary of papers published within this Research Topic. Generated using BioRender.com.

Autophagy (*self-eating*) is an evolutionary conserved process. It plays an important role in keeping cells clear of damaging insults by degrading damaged proteins and organelles as well as fighting against infections. Autophagy also provides energy during fasting or exercise through the lysosomal degradation of cellular components. Therefore, it is not surprising that autophagy is involved in protection against many diseases, including cancer (Mizushima and Levine, 2020). Multiple studies have investigated the role of autophagy in cancer development and progression showing that this cellular process could play onco-suppressive or oncogenic functions (Vega-Rubin-de-Celis, 2021). Thus, autophagy dysregulation has been proposed to contribute to cancer generation and progression by facilitating some of the events that lead to cell transformation. Likewise, autophagy stimulation could be one of the mechanisms by which primary or metastatic tumors adapt to the hypoxic and nutrient-deprived environment characteristic of the cancer niche. However, the precise mechanisms underlying these effects as well as the events that regulate the expression of key autophagy regulatory genes in cancer cells are far from being fully understood. Furthermore, several clinical trials in different tumor types have been developed using autophagy modulators (especially the non-specific autophagy inhibitor chloroquine) (Mohsen et al., 2022). Unfortunately, results from these studies have been disappointing, probably reflecting the need for a better understanding of the role of autophagy in tumorigenesis. This special issue includes a total of seven papers, including six original articles and one review that might help to improve our understanding of these processes (Figure 1).

The importance of autophagy-related non-coding RNAs in carcinogenesis is summarized in a review by de la Cruz-Ojeda et al. This manuscript discusses some of the mechanisms that could be involved in the regulation of the expression of autophagic genes during cancer generation and progression underlining the key role played by ncRNAs in the control of macroautophagy (de la Cruz-Ojeda et al.).

As discussed above, depending on the cellular context and the stage of tumor progression, activation or inhibition of autophagic flux will impact oncogenesis differently. Following this interesting question, one article included in this special issue explores the role of chaperone-mediated autophagy (CMA) in pericytes - one of the cell types that plays a crucial role in the glioblastoma microenvironment (Molina et al.). The possibility of selectively targeting macroautophagy or CMA in certain cell types is an issue of great interest that may have potential therapeutic implications.

Autophagy plays a dual role in the regulation of cell survival. Accordingly, promoting the activation of cytotoxic autophagy (autophagy-mediated cell death) has been a significant part of the work in the autophagy and cancer field. Likewise, avoiding the activation of protective autophagy as a mechanism of resistance to cancer therapies is an issue of utmost importance that has been the subject of intensive research. Three articles included in this issue of Frontiers in Cell and Developmental Biology explore these questions in further detail. Thus, in the article by Yang and colleagues (Yang et al.), the authors investigate the autophagic mechanism by which a natural compound activates cancer cell death. In their study, they demonstrated that TEOA induced autophagic cell death in pancreatic ductal adenocarcinoma cells by promoting mitochondria dysfunction and increasing ROS production. Another article (Gamez-Garcia et al.) identifies a new signaling mechanism involved in the regulation of autophagy-mediated cancer cell death that may have very interesting therapeutic implications. Briefly, ERK5 inhibition induces ER stress which leads in turn to autophagy-mediated cancer cell death. Finally, Hegedüs et al. identified a potential resistance mechanism to treatment with tyrosine kinase inhibitors in malignant pleural mesothelioma that is associated with autophagy stimulation. Interestingly, such a mechanism does not seem to rely on the regulation of the AKT/mTORC1 and ERK pathways (Hegedus et al.).

A third important aspect of autophagy research in the cancer field is focused on the investigation of the potential use of readouts of the autophagic process (including the expression of autophagy-associated genes) as potential biomarkers of cancer progression or response to therapy. Two articles within this Research Topic explore this question and, specifically, the potential connection between autophagy and the immune microenvironment (Wang et al.; Xu et al.).

Research in the autophagic field has yielded spectacular advances in the last couple of decades but there are still many burning questions that remain to be investigated and, specifically, in the context of the role of this cellular process in cancer. Altogether, studies published in the present topic highlight the importance of more precisely dissecting the mechanisms that regulate the activation of autophagy.
